# Inducing Physical Inactivity in Mice: Preventing Climbing and Reducing Cage Size Negatively Affect Physical Fitness and Body Composition

**DOI:** 10.3389/fnbeh.2019.00221

**Published:** 2019-10-04

**Authors:** Peter Roemers, Yasmin Hulst, Steffen van Heijningen, Gertjan van Dijk, Marieke J. G. van Heuvelen, Peter P. De Deyn, Eddy A. van der Zee

**Affiliations:** ^1^Molecular Neurobiology, Groningen Institute for Evolutionary Life Sciences (GELIFES), University of Groningen, Groningen, Netherlands; ^2^Behavioral Neuroscience, Groningen Institute for Evolutionary Life Sciences (GELIFES), University of Groningen, Groningen, Netherlands; ^3^Center for Human Movement Sciences, University of Groningen, University Medical Center Groningen, Groningen, Netherlands; ^4^Department of Neurology and Alzheimer Research Center, University of Groningen, University Medical Center Groningen, Groningen, Netherlands

**Keywords:** physical inactivity, sedentary behavior, rodent, climbing, cage size reduction, body composition, cognition, anxiety

## Abstract

Physical inactivity has emerged as an important and risk factor for cardiovascular and metabolic diseases, independent of levels of exercise engagement. Moreover, inactivity is associated with poor brain functioning. However, little data on the effects of physical inactivity on the brain is available and few methods are suitable to investigate this matter. We tested whether preventing lid climbing and reducing cage size could be used to model physical inactivity in mice. Sixty young adult C57Bl6 mice (10 weeks old) were divided over six groups with different housing conditions: in cages of three different sizes with lids that either allowed or prevented lid climbing. Housing under these conditions was maintained for a period of 19 weeks before the mice were killed for body composition analysis. Physical fitness tests performed around 5 and 10 weeks into the intervention revealed that motor coordination in the balance beam test was reduced by 30.65%, grip strength by 8.91% and muscle stamina in the inverted screen test by 70.37% in non-climbing mice as compared to climbing controls. Preventing climbing increased visceral fat mass by 17.31%, but did not reduce muscle mass. Neither preventing climbing nor reducing cage size affected anxiety assessed in the Open Field test and the Elevated Plus Maze. We did not find any negative effect of inactivity on spatial learning and memory in the novel object location test or working memory measured with the Y-maze Alternation test. The reduced physical fitness and increase in visceral fat mass show that our inactivity method models most effects of physical inactivity that are observed in experimental and observational studies in humans. Whereas established methods such as hindlimb unloading mimic many of the effects of bed rest, our novel method can be applied to study the effects of less extreme forms of physical inactivity (i.e., sedentary behavior) in various disease models including rodent models for brain diseases (i.e., stroke, Alzheimer’s disease).

## Introduction

The level of physical activity of humans in contemporary “western” societies is quite low as compared to that of their counterparts adhering to a hunter-gatherer lifestyle or a (more evolutionary recent) traditional agricultural lifestyle (Hayes et al., 2005; Katzmarzyk, 2010; Raichlen et al., 2017). Studies performed in an Inuit population in transition to this western modus operandi illustrate the detrimental influence of a sedentary lifestyle on aerobic fitness, strength and body mass (Rode and Shephard, 1994). In fact, physical inactivity is arguably one of the most important health problems of the 21st century (Guthold et al., 2018). In the past decades, physical inactivity was revealed to be a risk factor for a number of diseases including obesity, cardiovascular diseases, stroke, diabetes type 2 and colon cancer (Mansoubi et al., 2014; Biswas et al., 2015). Inactivity is also one of the largest preventable risk factors for Alzheimer’s disease in both Europe and the United States (Barnes and Yaffe, 2011; Norton et al., 2014).

Scientists and policymakers have often pointed towards exercise engagement as a possible measure to counteract the detrimental effects of physical inactivity. However, recent findings show that it is unlikely the risks that come with sedentary behaviors are simply caused by a lack of exercise. First, studies have revealed a dose-responsive relationship between sedentary behaviors (sitting, watching tv) and cardiovascular disease-related risk factors and mortality, which is independent of levels of exercise engagement (Hu et al., 2003; Katzmarzyk et al., 2009; Ekelund et al., 2016). Second, non-exercise activities such as standing and walking are associated with lowered metabolic risk and this association is independent of exercise levels (Healy et al., 2007, 2008). Third, breaking up prolonged sitting time with non-exercise movement will counter the deleterious effects of sitting on cardiovascular and metabolic parameters (Benatti and Ried-Larsen, 2015). In fact, non-exercise activity is likely to play a pivotal role in regulating energy expenditure and is negatively associated with obesity (Levine, 2004; Katzmarzyk, 2010).

Although some headway has been made, exercise-independent effects of physical inactivity on brain health and functioning have scarcely been investigated (Falck et al., 2017). The mechanisms *via* which physical inactivity affects the brain remain to be elucidated. Animal studies could be used to investigate these mechanisms. However, although various exercise models are available, few suitable methods that induce physical inactivity in rodents have been developed.

One method that is often used to study the unfavorable effects of physical inactivity in rodent models is hindlimb unloading (Morey-Holton and Globus, 2002; Cho et al., 2016). This method reduces the weight-bearing use of the hindlimb muscles for extended periods of time by suspending a rat or mouse from the ground by its tail or by using a harness. Hindlimb unloading was initially developed to mimic the weightlessness encountered during spaceflight but has successfully been adopted to investigate the effects of decreased use of the hindlimb muscles and inactivity physiology.

There are some serious downsides with regard to its usefulness in investigating the effects of inactivity on brain and behavior. First, hindlimb unloading causes acute stress, although stress levels may return to baseline on the long-term (Steffen and Musacchia, 1987; Hanson et al., 2013). This could influence brain biochemistry as well as cognitive functions, including learning and memory (Morey-Holton and Globus, 2002; Kim et al., 2006). Second, the associated muscle atrophy is quite severe (e.g., 25% normalized soleus mass in mice in Hanson et al., 2013). Due to its severity this may seriously confound the outcomes of subsequent behavioral tests that measure cognition but rely on basic locomotor activity of the mouse during walking, running or swimming. Third, the unloading method limits behavior of rodents in an extreme manner, as ambulatory movements are restricted. It is therefore impossible to distinguish between the direct effects of unloading and effects of restricted freedom in behavior. Another method to induce decreased use of skeletal muscle, immobilization of the animals’ leg joints and muscles by wrapping the legs with a plaster bandage, causes similar problems (Cho et al., 2016).

In summary, these methods have important strengths which have been employed to investigate the effects of inactivity on e.g., muscles and bones, but are less suitable to investigate brain and behavior. This is largely due to the fact that they are quite restrictive, not just to preventing muscle use but also aspects of behavior less relevant to inducing inactivity. In the current study, we tested novel, less restrictive methods to induce sedentary behavior in a laboratory setting in young adult mice and we explored the effects of induced inactivity on locomotor behavior, anxiety and learning and memory.

We aimed to model the effects of physical inactivity in mice by modifying their home cage and thereby their voluntarily executed ambulatory movement. Locomotor behavior in the home cage is already quite limited, but mice do climb in their lids and walk around in their cages. Climbing takes place almost exclusively in the dark phase and mostly in the first hour of the dark phase (Bains et al., 2018). Young adult C57Bl6 (13 weeks of age) mice were shown to climb their lids for about 250 s in the first hour of the dark phase (Harri et al., 1999; Bains et al., 2018). Lid climbing can be prevented by modifying the lids (see “Cage and Lid Adjustments” section). With regard to walking, in a pilot study we observed that adult mice housed in a standard (l × w × h; 33 × 15 × 13 cm) cage walk around 110 m per night and that a 50% reduction in floor size reduced this distance to 70 m (see Supplementary Figure S1). Thus, reducing physical activity by preventing climbing and decreasing cage size could be used as a method to induce physical inactivity. Studies in humans have shown sedentary behavior and physical inactivity to be associated with a decrease in strength and motor coordination, a decrease in lean muscle mass and an increase in abdominal fat mass (Krogh-Madsen et al., 2010; van der Velde et al., 2017a,b). Hence, we hypothesized that preventing climbing and reducing cage size reduce strength, muscle stamina and motor coordination in mice. Moreover, we expected the induced physical inactivity to reduce muscle mass and increase fat mass.

Finally, we assessed whether physical inactivity affected anxiety and cognition in mice. It is known that sedentary behavior and anxiety are associated, particularly in older adults (Vancampfort et al., 2018). Moreover, experimentally induced physical inactivity can increase anxiety in young adults within a week (Edwards and Loprinzi, 2016). Finally, there is some evidence that links physical inactivity to decreased cognition in humans (Falck et al., 2017). Therefore, we tested the effects of the physical inactivity on anxiety and spatial learning and memory. We expected inactivity to increase anxiety and negatively affect learning and memory.

## Materials and Methods

### Experimental Groups

**Figure 1:**
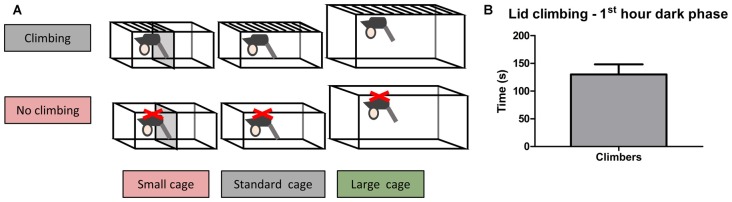
Home Cage adjustments and climbing behavior. Cage and lid adjustments. **(A)** Schematic overview of the various adjustments that were made to the lid and the cage. Mice were housed with a metal wire lid or with the same lid covered from underneath with a plastic sheet containing small holes. The covering of the lid was meant to prevent climbing. Furthermore, mice were housed in cages of various sizes (see “Cage and Lid Adjustments” section of “Materials and Methods”). **(B)** Lid climbing during the first hour of the dark phase for mice with wire lids (climbers), scored in week 7. No lid climbing was observed in mice housed with lids covered with a plastic sheet (non-climbers). Values are mean ± SEM, *n* = 30 climbers.

Adult male C57BL/6JRj mice (10 weeks old, *n* = 60) were purchased from Janvier. Mice were housed individually for 2 weeks before testing commenced, during this period the mice were weighed five times and handled four times to habituate them to handling. The mice were pseudo-randomly allocated to six housing conditions based on bodyweight and grip strength (as measured in week −2): Standard Cage with wire lid (climbers, *n* = 10) or adjusted lid (non-climbers, *n* = 10), Small Cage (50% reduction in size as compared to the standard cage) with wire lid (climbers, *n* = 10) or an adjusted lid (non-climbers, *n* = 10), and Large Cage (~twice the size of the standard cage) with wire lid (climbers, *n* = 10) or adjusted lid (non-climbers, *n* = 10; see Figure 1A, and see “Cage and Lid Adjustments” section).

### Animal Housing

Mice were individually housed in a temperature (22°C) and humidity (55%) controlled room, with *ad libitum* access to food and water. Individual housing was implemented to allow monitoring of physical activity and food intake of individuals. Small wood chips served as bedding material and shredded cardboard as nesting material. A 12:12 light/dark schedule was maintained (lights on at 06:00 h GMT). This study and all the procedures were approved by the ethical committee for the use of experimental animals of the University of Groningen.

### Cage and Lid Adjustments

Within our facility, it is standard procedure to place individually housed mice in a type 2 macrolon cage (l × w × h; 33 × 15 × 13 cm). This space was reduced for mice housed in small cages (l × w × h; 16.5 × 15 × 13 cm) by placing a plexiglass spacer in the middle of the cage. Mice housed in large cages were housed in a type 3 macrolon cage (l × w × h; 42 × 26 × 15 cm). To prevent climbing in the lid, a sheet of plastic containing small holes (3 mm ø) was placed under the standard wire lid and fixed using cable ties. This type of modification ensured light conditions were the same for climbers and non-climbers. A small piece of plastic was placed in all other wire lids, in the same manner, to control for possible licking or gnawing on the plastic. Because placing a food hopper would allow for climbing behavior on the hopper, food was placed directly in the cage for all groups and fresh food was provided two to three times a week based on a daily check. Finally, mice are able to latch on to the nozzle of their drinking bottle if it protrudes into the cage. In order to prevent this behavior in mice housed with adjusted lids, drinking bottles were placed so that the nozzle did not stick out.

**Figure 2:**
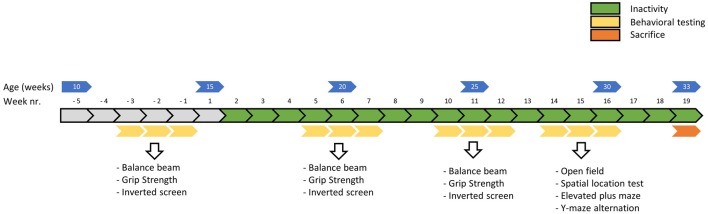
Timeline of the main experiment in weeks. Physical fitness tests were conducted before, around 5 weeks and around 10 weeks into the induced inactivity intervention. The balance beam test (week—3, 5 and 10) was conducted to asses motor skills, the grip strength test (week −2, 6 and 11) to assess strength and the inverted screen test (week—1, 7 and 12) to assess muscle stamina (or muscle fatigue). Around 15 weeks, behavioral tests were conducted to assess possible effects of induced inactivity on locomotor behavior, anxiety and cognition.

Climbing behavior was manually scored and observed using video recordings of the first hour of the dark phase using Observer behavior scoring software (Noldus Information Technology) in week 7 (see Figure 2).

### Behavioral Tests—Physical Fitness

Physical fitness tests were performed before, around 5 weeks and around 10 weeks into the period of physical inactivity (see Figure 2). All behavioral tests were conducted in the light phase, between 04:00 and 11:00 Zeitgeber time, in a dimmed light room that was located adjacent to the housing rooms (within one cluster).

#### Balance Beam Test

The balance beam test is a sensorimotor integration test used to test for differences in balance and motor skills. We adapted our protocol used to characterize exercise interventions by leaving out a particularly difficult round beam (Roemers et al., 2018). The balance beam test was performed before inducing inactivity (week—3) and after 5 and 10 weeks of physical inactivity. The balance beam apparatus consists of a rectangular wooden beam (4 mm width) placed 50 cm above a padded surface, with a safe cage at the end of the beam. This beam could be placed horizontally or under a 30° upward angle. During each session, three trials were performed with 30 s breaks, in between sessions there was a 90 s break. On the testing day, the animals were habituated to the task in two sessions using a 12 mm and 4 mm wide rectangular beam that was placed horizontally. Testing sessions were commenced directly (90 s break) after the habituation sessions. Time to cross 100 cm was measured during one session on an angled beam and subsequently during one session on a horizontally placed beam. We hypothesized that a decrease in strength would decrease performance on the angled beam more so than on the horizontal beams, as moving up at an angle demands more energy. Average time (s) to cross the beam was taken as an outcome measure.

#### Grip Strength Test

The grip strength test measures muscle force in mice *in vivo*. We used our protocol to characterize exercise interventions (Roemers et al., 2018). A validated grip strength meter (Columbus instruments) was used to test grip strength before inducing inactivity (week—2) and after 6 and 11 weeks of inactivity. The researcher performing the tests was blinded as mice were handed to him in a transport cage. Mice were picked up gently by their tails and allowed to grip on to a grid which was attached to the grip strength meter, then the mice were pulled backward horizontally by their tail until they let go of the grid. Peak force grams force (gf) was measured. First, forelimb grip strength was measured in four trials separated by 60 s breaks. After a 150 s break, all limb grip strength was measured in four trials separated by 60 s breaks. If a mouse did not hold on to the wired grid correctly, a retry trial was permitted twice within each session. Immediately after the test, the mice were weighed. The maximum peak force was taken as a measure for grip strength.

#### Inverted Screen Hanging Test

The inverted screen hanging tests was used to assess muscle stamina, or muscle fatigue, before inducing inactivity (week—1) and after 7 and 12 weeks of inactivity. We used the same protocol used to characterize exercise interventions (Roemers et al., 2018). The apparatus used was a wire grid (wires 2 mm ø) surrounded by a small wooden frame. Mice were placed on the grid, which was inverted and placed 50 cm above a padded surface. Four mice were tested at the same time in the same room. During the test, mice were separated so they could not see each other. Maximum hanging time was set to 20 min. If a mouse fell within 20 min, a second trial was performed after a 300 s break. Immediately after the test, the mice were weighed. The time until fall was scored using video recordings. Maximum time until fall (s) and “Holding Impulse” were used as output measures. The Holding Impulse is an output measure that corrects for variation in bodyweight and was calculated by multiplying body mass (g) by maximum time until fall (s).

### Behavioral Tests—Anxiety and Cognition

Behavioral tests for anxiety and cognition were performed in week 15, 16 and 17 of induced physical inactivity (see Figure 2). All behavioral tests were conducted in the light phase, between 04:00 and 11:00 Zeitgeber time, in a dimmed light room that was located next to the room where the animals were housed. The tests were performed in the order they are presented below.

#### Open Field Test

The open field test was used to test locomotor activity and can be used to assess anxiety in week 15. Before testing commenced, mice were habituated for 3 days by carrying them to the test room in groups of four and by handling them for 1 min. One day after the last habituation session, the mice were placed in an empty arena of 50 × 50 × 35 cm and allowed to explore the arena for 10 min. Light intensity was 11.91 lux in the middle, 9.91 lux on the sides and 8.35 lux in the corners of the boxes. Two mice were tested at the same time in adjacent arenas. The mice were filmed and their behavior was analyzed with Ethovision video tracking software (Noldus Information Technology). Distance traveled (cm) was taken as a measure for locomotor activity and the percentage of time spent in the center zone was taken as a measure for anxiety.

#### Spatial Location Recognition Test

Hippocampus-dependent spatial memory was tested with the spatial location recognition (SLR) test in week 15 and 16. This test took place 3 days after execution of the open field test and the mice were handled for 1 min in the experimental room on the days between the two tests. In a first session, mice were allowed to explore the empty test arena 41 × 30 × 30 cm for 10 min. Light intensity was 9.05 lux in the middle and 7.78 lux in the corners of the boxes. Subsequently, three training trials of 10 min each were conducted, during which three objects were placed in the box. One object was placed in the middle, two objects were placed in the corners. The animal was placed in the same box again during the test trial 24 h later. This time the location of one corner object was changed (to the opposite corner, the object in the middle was never moved). The box, the location of the objects during the training and the moved object were pseudo-randomly divided over the animals and groups. Boxes and objects were cleaned after each trial using 70% ethanol. All trials were filmed and the time the mouse spent exploring each object was scored using Observer behavior scoring software (Noldus Information Technology). Time spent exploring the moved object (corner) divided by the stationary object (corner) was taken as a measure for spatial memory.

#### Spontaneous Alternation Test

The spontaneous alternation test was used to test spatial working memory and was conducted minimally 3 days after the SLR test in week 17. The Y maze consisted of three arms spaced 120° from each other, each arm was evenly lighted (10 lux). The maze was placed in a room that contained spatial cues on the walls. Mice were placed in the middle of the maze and allowed to freely explore the maze for 8 min. The order in which the mouse visited the arms was scored and an alternation score was calculated to express the number of triads of unique arms visited (i.e., “A, B, C” or “B, C, A”, but not “A, A, B”). The alternation score was calculated as the total number of triads made expressed as a percentage of the maximum amount of triads possible [“ABCBACBA” gives five out of six possible triads (Anisman, 1975)].

#### Elevated Plus Maze Test

Anxiety was measured in the elevated plus maze test. The elevated plus maze consists of two open arms and two closed arms (length of the arms: 29.5 cm, center zone: 5 × 5 cm), and stands 50 cm above the ground. Mice are naturally averse to enter open spaces: less time spent in the open arms indicates higher anxiety. Mice were placed in the maze facing the center zone, so that they could choose to enter one of the closed arms immediately without having to turn around. Mice were allowed to explore the maze freely for 8 min. Video recordings were used to assess the time spent in the center or either of the arms using Ethovision video tracking software (Noldus Information Technology). Time spent in the open arms, expressed as a percentage of the total time spent in both closed arms and open arms, was taken as a measure for anxiety.

### Food Intake

Food intake was measured in week 13 and 14 of the inactivity intervention by weighing how much food was placed inside of the cage and by weighing how much food was taken out of the cage at the end of each week. After removal from the cage, the food was first stored in an open package for 6 days in order to evaporate any excess moisture.

### Tissue Collection and Weighing: Muscles, Fat Pads

In week 19, mice were killed *via* an intraperitoneal injection of pentobarbital. Food was rationed (2 g) during the dark-phase before the day of sacrifice, which effectively fasted the mice in the last 6 h of the dark phase. This was performed in order to do fasting glucose measurements, but the glucose level measurements that were obtained were probably affected by the pentobarbital (levels were very high, but there were no differences between groups, see Supplementary Table S1). Mice were transcardially perfused with a 0.9% NaCl + Heparin solution. Hindlimb muscles (M. Soleus, Gastrocnemius and Quadriceps Femoris), specific fat pads (i.e., epididymal fat, retroperitoneal fat, gonadal fat and inguinal) were dissected out and weighed. Blood samples were centrifuged (1,500 *g*, 15 min, 4°C) to collect a serum sample which was stored at −80°C.

### Body Composition Analysis

Dry and dry lean organ masses were determined by drying organs to constant mass at 103°C [ISO 6496-1983(E)] followed by fat extraction with petroleum ether (Boom BV, Meppel, NL, USA) in a soxhlet apparatus.

### Statistical Analyses

All data are presented as mean ± SEM. A value of *p* < 0.05 was considered as statistically significant, a value of *p* < 0.10 a trend for a difference. Partial eta squared ($\eta_{\text{p}}^{2}$) is given as a measure for effect size for mixed repeated measures analysis of variance (ANOVA) or two-way ANOVA, eta squared (*η*^2^) is given for one-way ANOVA and Cohen’s *d* is given for the one-sample *t*-test. The following benchmarks can be used as a rule of thumb to interpret effect sizes: For $\eta_{\text{p}}^{2}$ and *η*^2^: 0.01 = small effect, 0.06 = medium effect and 0.14 = large effect. For Cohen’s *d*: 0.2 = small effect, 0.5 = medium effect and 0.8 = large effect.

The effects of preventing climbing and modifying cage size on bodyweight and on the outcomes for the balance beam, grip strength test, inverted screen test and on bodyweight were analyzed using a mixed repeated measures ANOVA. In this analysis “climbing” (climbing, no climbing) and “cage size” (small, standard, large) were the between subjects factors and “time” (the three time-points)’ was the within subjects factor. If any significant main or interaction effect of the between-subjects factor or an interaction between time and either between-subjects factor was found, this analysis was followed by a two-way ANOVA to investigate the effect of “climbing” and “cage size” per time point and a *post hoc* test (Tukey-HSD) to determine the difference between cage sizes if a main effect of cage size was revealed. If the mixed repeated measures ANOVA or the Two-Way ANOVA showed a significant “climbing” by “cage size” interaction effect, a one-way ANOVA which included any of the six housing conditions (climbing/no climbing × cage size) as between subjects factors was performed per time point. This was followed by a *post hoc* test (Tukey-HSD) if there was a significant difference between any of the groups.

The effects of preventing climbing and cage size on food intake, fat mass, muscle mass, performance in the open field test, the elevated plus maze, the SLR test and the Y-maze alternation test were analyzed using two-way ANOVA and a *post hoc* test (Tukey-HSD) to determine the difference between cage sizes if a main effect of cage size was revealed. If the two-way ANOVA analysis showed a significant “climbing” by “cage size” interaction effect, a one-way ANOVA which included any of the six housing conditions (climbing/no climbing × cage size) as between subjects factors was performed. This was followed by a *post hoc* test (Tukey-HSD) if there was a significant difference between any of the groups.

## Results

### Climbing Behavior

In the first hour of the dark phase, mice climbed 130.0 ± 18.01 s in 9.86 ± 1.41 climbing bouts (see Figure 1B). Climbing was not observed in any of the mice housed in cages with adjusted lids.

### Physical Fitness Tests

#### Balance Beam Test

**Figure 3:**
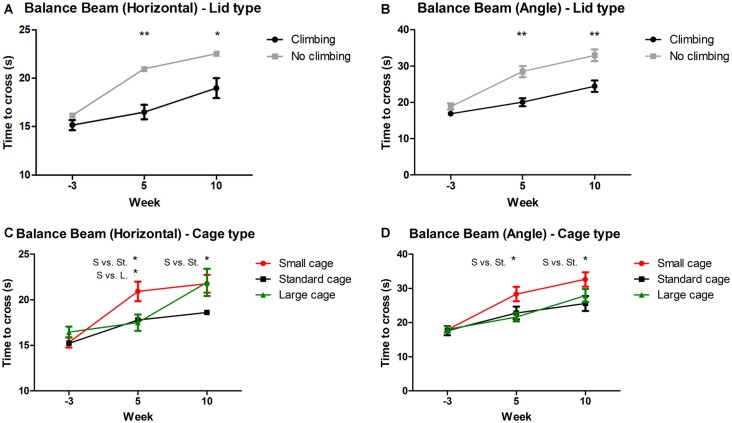
Balance beam performance. Balance beam performance was tested before (week—3) and after 5 and 10 weeks of induced inactivity. The mean time to cross over three trials was taken as a measure for motor coordination. **(A,B)** Preventing climbing significantly increased time needed to cross the balance beam whether placed horizontally or at a 30° angle. **(C)** Mice housed in smaller cages took longer to cross the horizontal balance beam as compared to mice housed in a large cage after 5 weeks, and as compared to mice housed in a standard cage after 5 and 10 weeks. **(D)** Mice housed in a smaller cage took longer to cross the balance beam placed at a 30° angle as compared to mice housed in a standard sized cage after 5 and 10 weeks. *,**indicate significant differences (*p*-values <0.05 and <0.01 respectively). “S vs. St.” and “S. vs. L.” indicate a difference between mice housed in Small and Standard cages and mice housed in Small and Large cages respectively. Values are mean ± SEM, *n* = 30 per groups for Climbing vs. No climbing and *n* = 20 per group for the comparison of different cage sizes (Small, Standard, Large).

Mixed repeated measures ANOVA showed that balance beam performance decreased over time points when all mice were grouped together (for both the angled and horizontal beam). This decline was present in all groups (see Figures 3A–D). Both preventing climbing (angle: *p* < 0.001, $\eta_{\text{p}}^{2}$ = 0.13; horizontal: *p* = 0.004, $\eta_{\text{p}}^{2}$ = 0.10) and decreasing cage size (angle: *p* = 0.008, $\eta_{\text{p}}^{2}$ = 0.19, horizontal: *p* = 0.001, $\eta_{\text{p}}^{2}$ = 0.15) worsened this decline over time. Two-Way ANOVA revealed no difference in balance beam performance at baseline (week—3) between any of the groups (climbing/no climbing × cage size), although there was a trend for a difference between mice that were to be housed as non-climbers and climbers when the beam was placed at an angle (*p* = 0.097, $\eta_{\text{p}}^{2}$ = 0.05). After 5 and 10 weeks of induced inactivity, non-climbers (angle: *p* < 0.001 and *p* < 0.001, horizontal: *p* < 0.001 and *p* = 0.016, see Figures 3A,B) and mice housed in a small cage [angle: *p* = 0.006 and 0.028, $\eta_{\text{p}}^{2}$ = 0.17 and $\eta_{\text{p}}^{2}$ = 0.12; horizontal: *p* = 0.018 and ns, $\eta_{\text{p}}^{2}$ = 0.13 (and ns), see Figures 3C,D] were significantly slower to cross the beam. The effect of housing mice in small cages was most clear on the angled beam (see Figure 3D). Pairwise comparisons revealed that mice housed in a small cage took longer to cross the beam as compared to mice housed in a standard cage in week 5 (angle: *p* = 0.032, horizontal: *p* = 0.048) and week 10 (angle: *p* = 0.024, horizontal: ns), and as compared to mice housed in a larger cage in week 5 (angle: *p* = 0.007, horizontal: *p* = 0.028) but not in week 10 (see Figures 3C,D). Although there was no significant interaction between climbing and cage-size, the effects of preventing climbing were most clear for the non-climbing animals housed in small cages (see Supplementary Table S1). For detailed data on all six housing conditions, see Supplementary Table S1.

On average, performance of non-climbers decreased by 30.65%: Non-climbers needed 22.89% more time to cross the horizontal beam (20.96 ± 0.84 and 22.54 ± 1.02 s to cross for non-climbers, vs. 16.50 ± 0.73 and 18.98 ± 1.03 s for climbers in week 5 and 10, respectively) and 38, 41% more time to cross the angled beam (28.47 ± 1.55 and 32.95 ± 1.59 s to cross for non-climbers, vs. 20.04 ± 1.09 and 24.45 ± 1.57 s for climbers in week 5 and 10, respectively).

#### Grip Strength

**Figure 4:**
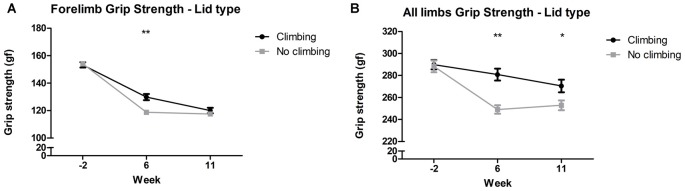
Grip strength test. The grip strength test was performed before (week—2) and after 6 and 11 weeks of induced inactivity. The maximum value out of all trials was taken as a measure for grip strength. **(A)** Preventing climbing significantly decreased forelimb grip strength in week 6. **(B)** Preventing climbing significantly decreased grip strength when the mouse could use all limbs in the test in week 6 and 11. *,**indicate significant differences (*p*-values <0.05 and <0.01 respectively). Values are mean ± SEM, *n* = 30 per group.

Mixed repeated measures ANOVA showed that forelimb and all limb grip strength declined over time, for both climbers and non-climbers (see Figures 4A,B). Preventing climbing worsened this decline over time for forelimb grip strength (*p* = 0.011, $\eta_{\text{p}}^{2}$ = 0.08) and all limb grip strength (*p* = 0.002, $\eta_{\text{p}}^{2}$ = 0.11). Cage size had no significant effect on grip strength. Two-way ANOVA revealed that after 6 but not 11 weeks of induced inactivity, forelimb grip strength was significantly decreased in non-climbers (*p* = 0.001, $\eta_{\text{p}}^{2}$ = 0.19, see Figure 4A). The effect of preventing climbing was most clear when grip strength was measured while the mouse held on with both fore- and hindlimbs (see Figure 4B). After both 6 and 11 weeks of induced inactivity, all limb grip strength was significantly decreased in non-climbers (*p* < 0.001 and *p* = 0.017, $\eta_{\text{p}}^{2}$ = 0.30 and $\eta_{\text{p}}^{2}$ = 0.10, see Figure 4B). After 11 weeks, two-way ANOVA revealed a trend for an effect of cage size on all limb grip strength (*p* = 0.07, $\eta_{\text{p}}^{2}$ = 0.09), which seemed attributable to a decline in grip strength in climbers housed in small cages (see Supplementary Table S1). When maximum grip strength was divided by bodyweight, similar effects were found (Supplementary Table S2). For detailed data on all six housing conditions, see Supplementary Table S1.

All limb grip strength was decreased by 8.91% in non-climbing mice (249.04 ± 3.82 and 252.92 ± 4.45 gf for non-climbers, vs. 280.82 ± 5.37 and 270.49 ± 5.71 gf for climbers in week 6 and 11, respectively).

#### Inverted Screen Test

**Figure 5:**
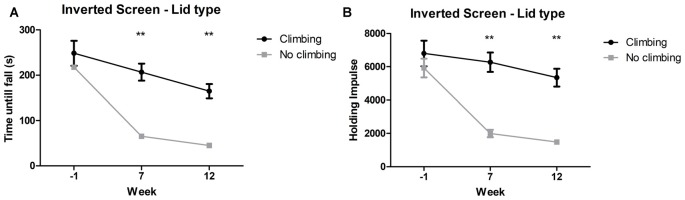
Inverted screen test. The inverted screen test was performed before (week—1) and after 7 and 12 weeks of induced inactivity. The maximum value out of two trials was taken as a measure for muscle stamina. **(A)** Preventing climbing significantly decreased hanging time and **(B)** holding impulse [max hanging time (s) × bodyweight (g)]. **indicates a significant difference (*p*-value <0.01). Values are mean ± SEM, *n* = 30 per group.

Mixed repeated measures ANOVA showed maximum time to fall and holding impulse (time × bodyweight) declined over time, for both climbers and non-climbers (see Figures 5A,B). Preventing climbing worsened this decline over time for both outcome measures (*p* < 0.001 and *p* < 0.001, $\eta_{\text{p}}^{2}$ = 0.15 and $\eta_{\text{p}}^{2}$ = 0.19). Cage size had no significant effect on performance in the inverted screen test. Two-way ANOVA revealed no differences between groups for either outcome parameter at baseline (week −1). After 7 and 12 weeks, time until fall (*p* < 0.001 and *p* < 0.001, $\eta_{\text{p}}^{2}$ = 0.47 and $\eta_{\text{p}}^{2}$ = 0.48) and holding impulse (*p* < 0.001 and, $\eta_{\text{p}}^{2}$ = 0.47 and $\eta_{\text{p}}^{2}$ = 0.46) were decreased in non-climbers (see Figure 5). For detailed data on all six housing conditions, see Supplementary Table S1.

Time until fall was reduced by 70.51% in non-climbers (65.30 ± 7.37 and 45.10 ± 5.10 s for non-climbers, vs. 206.73 ± 18.820 and 164.73 ± 15.88 s for climbers in week 7 and 12, respectively). Holding Impulse was reduced by 70.23% in non-climbers (1,993.81 ± 223.30 and 1,482.80 ± 153.20 for non-climbers, vs. 6,271.35 ± 583.76 and 5,345.25 ± 539.40 for climbers in week 7 and 12, respectively).

### Food Intake and Body Composition

Preventing climbing increased fat mass, decreased food intake and tended to increase bodyweight (see below). On the other hand, cage size did not significantly affect food intake, bodyweight and composition, muscle mass or fat mass (see Tables s 1, 2). Therefore, the following sections will mainly discuss the effects of preventing climbing. Importantly, some of the effects of preventing climbing do seem mainly attributable to the non-climbing mice housed in a small cage (see “Food Intake and Bodyweight” and “Fat Mass” sections).

**Table 1:** Bodyweight, tissue mass and body composition.

Group		Climbing	Cage size	Climbing × Cage size	Small		Small cage–No climbing		Standard cage		Standard cage–No climbing		Large cage		Large cage–No climbing	
					Mean	SEM	Mean	SEM	Mean	SEM	Mean	SEM	Mean	SEM	Mean	SEM
Bodyweight (g)	Wk-4	ns	ns	ns	25.07	0.31	24.69	0.40	25.55	0.13	24.26	0.57	24.83	0.39	25.41	0.20
	Wk 19	#	ns	ns	34.56	1.00	37.12	1.13	35.03	1.06	35.96	0.81	35.11	0.86	35.50	0.69
Hindlimb muscles (mg)	*Quadriceps femoris*	ns	ns	ns	191.64	5.58	190.80	5.41	185.41	8.80	174.01	7.76	191.07	7.74	188.30	8.68
	*Quadriceps femoris*/bw	ns	ns	ns	5.60	0.28	5.18	0.21	5.37	0.37	4.87	0.29	5.45	0.24	5.33	0.29
	*Gastrocnemius*	ns	ns	ns	157.47	3.21	158.44	1.62	159.26	4.22	152.91	3.32	162.28	2.10	163.71	5.33
	*Gastrocnemius*/bw	ns	ns	ns	4.59	0.18	4.30	0.12	4.59	0.21	4.27	0.13	4.64	0.12	4.64	0.22
	*Muscle soleus*	ns	ns	*	9.46	0.31	9.17*^a^	0.18	10.46*^B^	0.43	9.53	0.27	8.79*^b^	0.51	10.91*^A^**^B^	0.53
	*Muscle soleus*/bw	ns	ns	*	0.27	0.01	0.24*^A#B^	<0.00	0.30^#b^	0.01	0.26	<0.00	0.25^#C^	0.01	0.30*^a^	0.01
Lean mass carcass (after fat extraction)	g	ns	ns	ns	3.09	0.05	3.09	0.04	3.18	0.07	3.05	0.05	3.05	0.06	3.13	0.04

Effects of physical inactivity on bodyweight and muscle tissue mass. Absolute values or values divided by bodyweight (indicated by “/bw”). Main statistical analysis is summarized in the 3rd, 4th and 5th column (two-way ANOVA). Capital and lower case letters indicate differences between any of the six groups (one-way ANOVA). *,**indicate a significant difference (*p*-values < 0.05 and < 0.01 respectively), a “#” indicates a trend for a difference (*p*-value < 0.10). An “ns” indicates no significant differences were found. Group sizes are as follows: *n* = 30 per group for Climbing vs. No climbing and *n* = 20 per group for the comparison of different cage sizes (Small, Standard, Large), and *n* = 10 for each of the six distinct housing conditions.

**Table 2:** Fat pad mass and body composition.

Group		Climbing	Cage size	Climbing × Cage size	Small		Small cage–No climbing		Standard cage		Standard cage–No climbing		Large cage		Large cage–No climbing	
					Mean	SEM	Mean	SEM	Mean	SEM	Mean	SEM	Mean	SEM	Mean	SEM
Visceral fat pads (mg)	Epididymal	*	ns	ns	1,391.41	155.23	1,888.19	207.58	1,478.50	187.58	1,693.82	108.13	1,494.17	121.05	1,567.58	86.74
	Retroperitoneal	*	ns	ns	401.65	37.08	560.16	47.53	415.33	49.83	505.72	34.14	421.00	31.73	440.58	22.41
	Perirenal	ns	ns	ns	280.44	35.06	332.42	35.94	280.14	26.74	280.31	19.89	263.53	18.87	269.83	21.61
	Epi + Retro + Peri	*	ns	ns	2,073.50	222.99	2,780.77	272.78	2,173.97	250.33	2,479.85	154.82	2,178.70	163.96	2,277.99	116.43
Subcutaneous fat	Inguinal	*	ns	ns	738.52	82.68	996.41	118.84	722.85	76.70	878.62	60.50	702.64	60.40	703.46	40.36
pads (mg)
Visceral fat	Liver	ns	ns	ns	83.87	9.54	82.33	10.08	71.97	13.00	92.28	7.84	68.30	8.19	78.20	8.69
extraction (mg)
	Liver/bw	ns	ns	ns	2.45	0.30	2.19	0.24	2.03	0.35	2.56	0.21	1.96	0.24	2.21	0.25
	Gut	*	ns	ns	770.27	64.26	1,026.17	109.48	902.12	61.93	937.47	60.92	851.96	67.14	936.98	40.16
	Gut/bw	*	ns	ns	22.05	1.38	27.15	2.16	25.58	1.16	25.87	1.28	24.08	1.45	26.35	0.87
Fat extraction (mg)	Skin	*	ns	ns	1,954.13	233.09	2,767.43	258.49	2,108.42	272.77	2,644.49	270.81	2,054.78	213.59	2,012.44	99.84
	Skin/bw	*	ns	ns	55.51	5.50	73.46	4.68	58.96	6.22	72.66	6.29	58.16	5.28	56.50	2.05
	Carcass	*	ns	ns	1,784.62	152.16	2,447.08	194.72	1,880.60	162.26	2,314.39	163.54	2,022.36	163.06	1,968.15	128.61
	Carcass/bw	*	ns	ns	50.92	3.19	65.22	3.51	53.00	2.84	63.74	3.31	56.96	3.42	55.21	3.07

Effects of physical inactivity on adipose tissue mass and body composition. Absolute values or values divided by bodyweight (indicated by “/bw”). Main statistical analysis are summarized in the 3rd, 4th and 5th column (two way ANOVA). Capital and lower case letters indicate differences between any of the six groups (one-way ANOVA). *indicates a significant difference (*p*-value < 0.05). An “ns” indicates no significant differences were found. Group sizes are as follows: *n* = 30 per group for Climbing vs. No climbing and *n* = 20 per group for the comparison of different cage sizes (Small, Standard, Large), and *n* = 10 for each of the six distinct housing conditions.

#### Food Intake and Bodyweight

**Figure 6:**
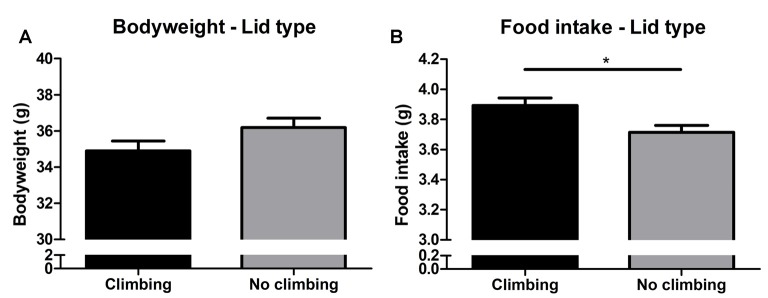
Bodyweight and food intake. **(A)** Bodyweight in week 19. There was a trend for preventing climbing to increase bodyweight over time as measured over all time points and analyzed using repeated measures analysis of variance (ANOVA; *P* = 0.06). **(B)** Preventing climbing significantly reduced food intake. *indicates a significant difference (*p*-value < 0.05). Values are mean ± SEM, *n* = 30 per group.

Bodyweight increased over time and there was a trend for further increase in bodyweight in non-climbers (*p* = 0.066, $\eta_{\text{p}}^{2}$ = 0.47, see Figure 6A). Although there was no significant interaction between climbing and cage-size, the effects of preventing climbing on bodyweight were mainly attributable to the non-climbing animals housed in smaller cages (see Table 1). Interestingly, despite this trend for increased bodyweight, non-climbers ate less than climbers (*p* = 0.010, $\eta_{\text{p}}^{2}$ = 0.11, see Figure 6B).

#### Fat Mass

**Figure 7:**
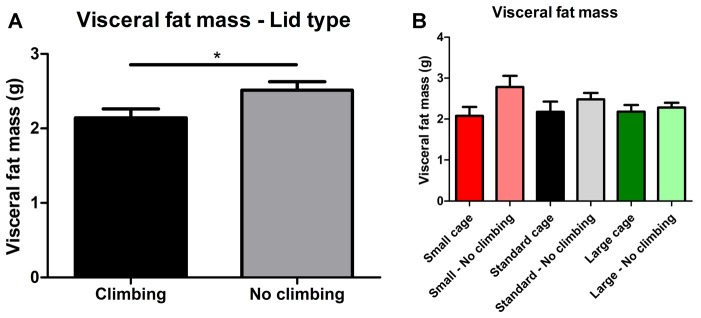
Visceral fat mass. Visceral fat mass was determined by adding the weight of three main visceral fat pads: the retroperitoneal, epididymal and perirenal fat pads. **(A)** Preventing climbing significantly increased visceral fat mass. **(B)** Although there was no significant interaction between preventing climbing and cage size, this increase in visceral fat mass by preventing climbing seemed mainly attributable to an increase in fat mass in non-climbing mice housed in small and standard sized cages. *indicates a significant difference (*p*-value < 0.05). Values are mean ± SEM, *n* = 30 per group for Climbing vs. No climbing and *n* = 10 per group for the six different groups that were differentiated by cage size and climbing/no climbing conditions.

Intra-abdominal fat mass was measured by means of fat extraction from the liver and gut as well as weighing of three main white adipose tissue pads located in the abdominal cavity (retroperitoneal, epididymal and perirenal fat pads). Gut fat mass was increased in non-climbers (*p* = 0.034, $\eta_{\text{p}}^{2}$ = 0.08, divided by bodyweight: *p* = 0.035, $\eta_{\text{p}}^{2}$ = 0.08). Liver fat mass did not differ between any of the groups. The combined weight of the three main visceral fat pads was significantly increased in non-climbers (*p* = 0.031, $\eta_{\text{p}}^{2}$ = 0.08, see Figure 7A). Visceral fat mass was increased by 17.31% in non-climbers (2,512.87 ± 114.24 mg for non-climbers, vs. 2,142.05 ± 120.37 mg for controls). Although there was no significant interaction between climbing and cage-size, the effects of preventing climbing were mainly attributable to the non-climbing animals housed in smaller cages (see Figure 7B, Table 2). The increase in visceral fat pad weight was caused by an increase in both epididymal and retroperitoneal fat pad mass (*p* = 0.038 and *p* = 0.006, $\eta_{\text{p}}^{2}$ = 0.07 and $\eta_{\text{p}}^{2}$ = 0.13, see Table 2), preventing climbing did not affect perirenal fat pad mass significantly.

Subcutaneous fat mass was estimated by fat extraction from the skin and weighing of the inguinal fat pad. Both skin fat mass (*p* = 0.026, $\eta_{\text{p}}^{2}$ = 0.08 divided by bodyweight: *p* = 0.022, $\eta_{\text{p}}^{2}$ = 0.09) and inguinal fat pad mass (*p* = 0.033, $\eta_{\text{p}}^{2}$ = 0.08) were significantly increased in non-climbers (see Table 2). Again, the effects of preventing climbing were mainly attributable to the non-climbing animals housed in smaller cages (see Table 2).

#### Muscle Mass

Muscle mass was measured by means of weighing three muscles in the hindlimb of the mice as well as by determining the lean mass of the carcass of the mice after fat extraction. Preventing climbing did not affect the mass of the (mainly) anaerobic gastrocnemius and rectus femoris muscle, whether corrected for bodyweight or not. There was a significant interaction between preventing climbing and modifying cage size on the mass of the (mainly) aerobic soleus muscle (*p* = 0.001, $\eta_{\text{p}}^{2}$ = 0.23 divided by bodyweight: *p* = 0.002, $\eta_{\text{p}}^{2}$ = 0.20). One-Way ANOVA revealed a significant difference between the six groups whether soleus weight was corrected for body mass (*p* = 0.011, *η*^2^ = 0.23) or not (*p* = 0.003, *η*^2^ = 0.23), *post hoc* comparisons (Tukey-HSD) revealed that non-climbers housed in a large cage had higher soleus muscle mass as compared non-climbers housed in a small cage or climbers housed in a large cage (*p* = 0.035 and *p* = 0.005, divided by bodyweight: *p* = 0.039 and *p* = 0.059). However, there were no clear main effects of preventing climbing or modifying cage size (see Table 1). Neither preventing climbing nor modifying cage size affected carcass lean mass (see Table 1).

### Anxiety or Cognition

#### Open Field Test and Elevated Plus Maze

Neither preventing climbing nor modifying cage size affected locomotor performance or behavior in the open field significantly. However, there was a trend for non-climbers to cover less distance in the open field test (*p* = 0.057, $\eta_{\text{p}}^{2}$ = 0.06) and to walk with a lower mean (*p* = 0.056, $\eta_{\text{p}}^{2}$ = 0.06) but not maximum velocity (see Table 3).

**Table 3:** Anxiety and cognition.

Group		Climbing	Cage size	Climbing × Cage size	Small		Small cage–No climbing		Standard cage		Standard cage–No climbing		Large cage		Large cage–No climbing	
					Mean	SEM	Mean	SEM	Mean	SEM	Mean	SEM	Mean	SEM	Mean	SEM
Y-maze	Alternation score	ns	ns	ns	67.85	3.76	60.18	3.74	62.27	3.66	62.42	4.30	58.67	4.22	66.19	3.26
	Entries	ns	ns	ns	26.60	2.36	24.60	0.90	27.50	3.18	24.60	1.59	23.10	2.53	23.60	1.28
Open field	Distance (cm)	#	ns	ns	2,735.73	228.60	2,905.67	194.62	3,459.93	298.13	2,847.24	174.05	3,219.37	327.78	2,519.16	167.81
	Time in center (%)	#	#	*	7.60	1.52	8.55	0.73	10.66*^a^	1.09	6.00*^A^	0.74	6.57	1.28	5.80*^a^	1.11
	Velocity (mean)	#	ns	ns	4.56	0.38	4.84	0.32	5.76	0.49	4.74	0.28	5.36	0.54	4.20	0.27
Elevated Plus Maze	Time spent in open arms (%)	ns	ns	ns	5.04	1.17	5.90	1.44	7.13	2.23	6.72	2.00	4.83	1.10	6.12	2.08
	Arm entries	ns	ns	ns	25.40	2.26	26.50	1.03	30.30	3.01	28.70	1.71	27.70	1.67	26.44	2.38
Spatial location recognition	Exploration moved object/exploration time unmoved corner object	ns	ns	ns	1.27	0.30	1.52	0.26	1.14	0.24	1.36	0.24	1.20	0.17	1.30	0.29
	Total exploration time (s)	ns	ns	ns	35.77	5.92	38.29	5.22	33.21	1.95	35.50	2.44	38.21	4.06	35.25	3.50

Effects of physical inactivity on anxiety and cognition. Main statistical analysis are summarized in the 3rd, 4th and 5th column (two way ANOVA). Capital and lower case letters indicate differences between any of the six groups (one-way ANOVA). An “*” indicates a significant difference (*p*-value < 0.05), a “#” indicates a trend for a difference (*p*-value < 0.10). An “ns” indicates no significant differences were found. Group sizes are as follows: *n* = 30 per group for Climbing vs. No climbing and *n* = 20 per group for the comparison of different cage sizes (Small, Standard, Large), and *n* = 10 for each of the six distinct housing conditions. For the elevated plus maze analysis, one outlier was excluded from “Small-no climbing” (see “Spontaneous Alternation and Novel Location Recognition Test” section).

**Figure 8:**
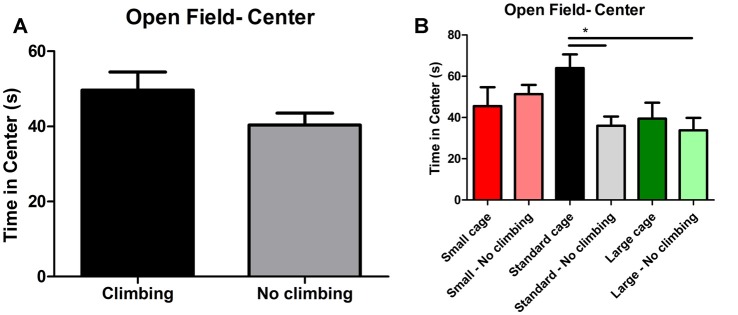
Open Field test. **(A)** Preventing climbing did not significantly decrease time spend in the center of the open field, but there was a trend for this effect. **(B)** There was a significant interaction between preventing climbing and cage size and non-climbers housed in a standard and large cage spend significantly less time in the center of the open field. *indicates a significant difference (*p*-value < 0.05). Values are mean ± SEM, *n* = 30 per group for Climbing vs. No climbing and *n* = 10 per group for the six different groups that were differentiated by cage size and climbing/no climbing conditions.

**Figure 9:**
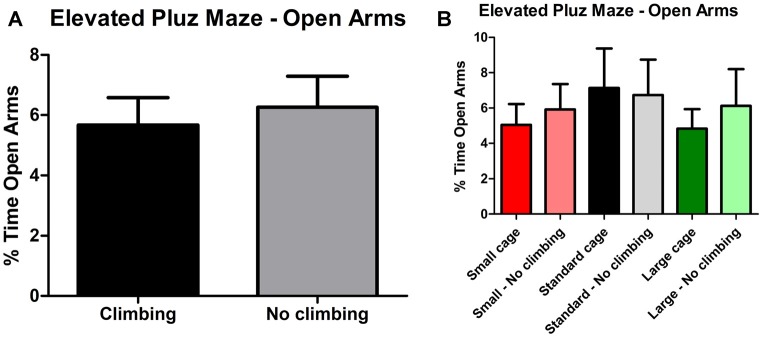
Elevated Plus Maze. **(A)** Preventing climbing did not significantly affect time spend in the open arms. **(B)** There was no difference between any of the groups as differentiated by cage size and climbing/not climbing conditions. Values are mean ± SEM, *n* = 30 per group for Climbing vs. No climbing and *n* = 10 per group for the six different groups that were differentiated by cage size and climbing/no climbing conditions.

Anxiety was assessed by measuring time spent in the center of the open field as well as by measuring time spent in the open arms in the elevated plus maze. There was a trend for non-climbers to spend less time in the center of the open field (*p* = 0.091, $\eta_{\text{p}}^{2}$ = 0.05, see Figure 8A). Moreover, there was a trend for an effect of cage size (*p* = 0.095, $\eta_{\text{p}}^{2}$ = 0.08) and a significant interaction between preventing climbing and cage size (*p* = 0.042, $\eta_{\text{p}}^{2}$ = 0.11) for time spent in the center. One-way ANOVA (*p* = 0.021, $\eta_{\text{p}}^{2}$ = 0.21) and Tukey HSD *post hoc* tests revealed that non-climbers housed in a standard or large cage spent less time in the center of the open field as compared to climbers housed in a standard cage (*p* = 0.045 and *p* = 0.024, see Figure 8B). However, neither preventing climbing nor modifying cage size affected time spent in the open arms in the elevated plus maze test (see Figure 9 and Table 3). One outlier was excluded from the elevated plus maze analysis: this mouse, part of the “small cage—no climbing” group, remained in one of the open arms for almost the entire test and hardly moved between arms.

#### Spontaneous Alternation and Novel Location Recognition Test

Neither preventing climbing nor modifying cage size affected spatial working memory as measured in the y-maze Spontaneous Alternation test (see Table 3).

One-sample *t*-test revealed that overall, mice explored the moved object more so than the unmoved object “exploration time moved object (corner)/exploration time unmoved corner object” was tested against chance level (against value: 1, *p* = 0.04, Cohen’s *d* = 0.38). However, neither preventing climbing nor modifying cage size affected spatial learning and memory in the novel location test (see Table 3).

## Discussion

Novel methods to induce inactivity in rodents are needed to investigate the (exercise independent) effects of physical inactivity on the brain and cognition. In the current study, we induced physical inactivity in mice by preventing climbing and by decreasing the size of the home cage. We investigated the effects of inducing physical inactivity on physical fitness, body composition, and anxiety and cognition in young adult mice.

### Preventing Climbing and Reducing Cage Size as a Novel Physical Inactivity Model

Preventing climbing reduced physical fitness as measured in three different behavioral tests: motor coordination on the balance beam, muscle strength in the grip strength test and muscle stamina (or muscle fatigue) in the inverted screen test. Reducing cage size further reduced motor coordination in the balance beam test, but did not affect grip strength or muscle stamina. Preventing climbing also tended to increase body mass and increased visceral and subcutaneous fat mass, even though food intake was reduced in mice that could not climb. However, induced inactivity did not affect cognition (see “No Negative Effects of Induced Physical Inactivity on Anxiety or Cognition in Young Mice” section).

#### Translational Value of the Inactivity Model

In humans, objectively measured sedentary time is associated with decreases in motor function and balance (walking tests), strength and cardiorespiratory fitness in adults and middle-aged adults, independently of exercise engagement (Kulinski et al., 2014; van der Velde et al., 2017a,b). Although we did not assess cardiorespiratory fitness or comparable measures in mice, our novel method successfully models the associations between sedentary behavior and reduced motor coordination and muscle strength in humans.

With regard to our physical fitness tests, two observations merit further discussion. First, a decline in performance in the physical fitness tests over the course of the experiment: performance decreased in the second and third bout of physical fitness tests independent of cage size or climbing opportunities. We encountered this issue before in an exercise study highly similar in set-up (Roemers et al., 2018). It seems unlikely that the decline in physical fitness is an effect of aging, since other studies do not see this decline in performance until mice are much older (>1 year of age; De Luca et al., 2003; Heng et al., 2007; Keeling et al., 2007; Brooks et al., 2012; Ge et al., 2016) Second, differences between inactive and control mice are more pronounced in the second time point as compared to the third. Although it seems unlikely, this suggests the effect of inducing physical inactivity on physical fitness becomes smaller over time. It seems more likely that habituation effects may have confounded results in our physical fitness measurements, although we did not observe any aberrant behavior during the tests. This could explain both the decline in performance over time and the decline in difference between groups, as performance in all three tests is dependent on negative reinforcers. Comparisons between the various housing conditions remain valid and support a detrimental effect of induced inactivity on physical fitness, but clearly the use of physical fitness tests could be optimized in future studies.

Observational studies in humans have also shown that sedentary behavior is associated with increased waist circumference, fat mass and cardiovascular risk factors independent of physical activity (Healy et al., 2007, 2008, 2011; Myers et al., 2017; Whitaker et al., 2017). Although these types of studies provide valuable information, observational studies cannot prove causal effects of sedentary behavior on body composition. Interestingly, two relatively small studies investigated the effects of a voluntary reduction of (non-exercise related) physical activity in humans in an experimental manner: young adult males that led physically active lives (6,000–10,000 steps per day) but did not exercise regularly were asked to refrain from rigorous physical activity and to reduce their daily activity to <1,500 steps per day. After 2 weeks of physical inactivity, the participants had developed metabolic impairments in glucose and fat tolerance tests, decreased aerobic fitness (a lower VO2max) and an increase in abdominal fat mass (accompanied by a decrease in total fat-free mass and BMI; Krogh-Madsen et al., 2010).

Although we did not observe a decrease in lean mass in our mice, the tendency for increased bodyweight in non-climbers and the increase in visceral fat mass we observed in our mice are in line with the increase in abdominal fat mass pointed out by these studies performed in humans.

### A Comparison to Established Models of Inactivity

Recently, the idea of sedentary behavior as an important risk factor which is independent of exercise has been gaining attention, but the subject has been studied for some time in the context of bed rest studies and rodent models that were used to mimic microgravity (primarily hindlimb unloading). These methods have some limitations with regard to the study of sedentary behavior that were partially touched upon in the introduction: in bed rest studies and the hindlimb unloading method restriction of movement and restriction of normal ambulatory behavior is quite extreme. This could affect behavior and physiology independent of the physical inactivity component of these methods. Moreover, the postural changes induced by either method could affect physiology in ways that do not relate to physical inactivity or sedentary behavior *per se*. Therefore a comparison between these methods and the method described in the current study should be made with caution. Another major difference between the current study and studies of either bedrest in humans and hindlimb unloading in rodents is the length of the intervention: physical inactivity was induced for a total period of 19 weeks in the current study, whereas bed rest interventions and hindlimb unloading interventions usually do not last longer than 1–4 weeks.

Keeping the above mentioned differences in mind, it is still interesting to compare the effects of these established models of physical inactivity to the method proposed in the current study. First, bed rest in humans, as well as hindlimb unloading in rodents, causes muscle atrophy (Bergouignan et al., 2011; Hanson et al., 2013; Bederman et al., 2015). Moreover, both bed rest in humans and hindlimb unloading in rodents decrease muscle strength (Stodieck et al., 2012; Dirks et al., 2016). Our novel method did not cause muscle atrophy, likely because animals were still able to perform light ambulatory movements. However, we did find that preventing climbing affected grip strength and muscle stamina. Second, hindlimb unloading is also associated with a decrease in bodyweight. This is caused by the atrophy, but also by atrophy of (visceral) adipose tissue (Bederman et al., 2015). In humans, bed rest can lead to a decrease in subcutaneous fat mass, but bed rest does not cause changes in visceral fat mass (Dirks et al., 2016). Preventing climbing actually increased visceral fat mass, something which is more in line with the observational and experimental studies that investigated the effects of sedentary behavior as outlined in the “Preventing Climbing and Reducing Cage Size as a Novel Physical Inactivity Model” section above (Healy et al., 2008, 2011; Krogh-Madsen et al., 2010; Whitaker et al., 2017). Finally, bed rest and hindlimb unloading cause insulin resistance (Bergouignan et al., 2011). This association between sedentary behavior and glucose tolerance has been described in studies that monitored or induced sedentary behavior in humans too (Healy et al., 2007; Krogh-Madsen et al., 2010). Since we did not investigate glucose tolerance by means of an oral glucose tolerance test, it is not yet known whether our novel inactivity model affects glucose tolerance.

Recently, another group published a novel method to model bed rest in rats by means of immobilization due to adjustments of the home cage (Marmonti et al., 2017). Cage volume was reduced to the extent that the animals could hardly move around and the lid did not enable them to climb, rats were confined to their home cage for 4 weeks. As this method was developed to model bed rest studies, an important difference with our study was the more extreme reduction of ambulatory activity. However, the method is less restrictive than available methods in the sense that the animals need not be handled or attached to any kind of apparatus. As in our model, immobilization decreased grip strength and food intake. Contrary to our model, immobilization did not increase fat mass and but did decrease soleus muscle mass (Marmonti et al., 2017).

### A Comparison to Exercise Models

It is noteworthy that the decrease in balance beam performance, decrease in grip strength and increase in (epididymal) fat mass that were caused by preventing climbing and (to a lesser extent) by decreasing cage size are of an opposite nature to the changes caused by various exercise models we characterized before (Roemers et al., 2018). A detailed comparison is beyond the scope of this article, but in many regards our physical inactivity method and exercise methods seem to target similar tissues and processes but in an opposite direction, as might have been anticipated.

### No Negative Effects of Induced Physical Inactivity on Anxiety or Cognition in Young Mice

Physical inactivity has been linked to decreased cognitive function and decreased mental health in older adults, but the relationship between physical inactivity and cognition has not been researched intensively. Moreover, depending on the type of sedentary behavior that is investigated, these associations may differ (Hamer and Chida, 2009; Hamer and Stamatakis, 2014; Falck et al., 2017). In the current study, we found no effects of induced physical inactivity on learning and memory in the spatial location test or Y-maze alternation test, nor clear effects on anxiety in young healthy male mice.

We included two standardized tests that investigate spatial learning and memory (novel location test) and spatial “working” memory (spontaneous alternation test). These two relatively basic tests revealed no negative impact of induced inactivity on spatial learning and memory. However, more elaborate tests (e.g., the Morris Water Maze, fear conditioning or inhibition dependent tasks) that address various aspects of learning and memory should be employed to assess this matter further and in more detail. Moreover, effects on cognition could arise when inactivity is induced at older age or after longer periods of physical inactivity.

Time spent in the center of the open field was reduced in mice housed in a large cage independent of climbing conditions and in mice that could not climb and were housed in a standard sized cage. Thus, housing conditions affect behavior in the Open Field test: we also found a trend for a decrease in distance traveled in non-climbers. But given that we found no differences in time spent in the open arm of the elevated plus maze, we cannot conclude that this is a reflection of increased anxiety *per se*. Thus, it is more likely that preventing climbing affects locomotor behavior in mice.

### Limitations and Recommendations

Although the current study successfully characterizes functional and structural effects of induced physical inactivity by preventing climbing and decreasing cage size, it is preliminary in nature. Therefore, it has several important limitations which have not yet been touched upon above. First, although gross indicators of muscle mass and changes in adipose tissue were investigated, we did not perform detailed analysis of muscle fiber adaptation or changes in for example numbers or size of fat cells (considered beyond the scope of this study). Second, the current study was performed in male mice only. Clearly, further characterization in female mice would be a welcome additional study. We have observed that climbing behavior seems just as readily performed by female mice of the same age. Finally, the current study made use of a limited number of mice per group (*n* = 10). Although comparisons between cage size (*n* = 20 each) and climbing vs. no climbing (*n* = 30 each) was performed in larger groups, the interaction effects between these two factors may have been harder to detect (for example the increase in visceral fat mass which was mainly observed in mice that could not climb and were housed in small or standard, but not large cages (see Figure 7B).

### Conclusion

Sedentary behavior has only recently been recognized as a risk factor and process which has exercise-independent effects. Much remains to be discovered about the effects of physical inactivity. The current study is preliminary in this regard, but shows that preventing climbing can be used as a model to further investigate the effects of physical inactivity in mice. In addition, decreasing cage size further affected motor coordination. Moreover, the increase in bodyweight and fat mass seemed most pronounced in non-climbing mice housed in a small cage (see Tables s 1, 2). Thus, preventing climbing while housing mice in a small cage is the most suitable method to induce physical inactivity.

### Perspectives

Inactivity methods such as hindlimb unloading have proven very valuable in investigating the more extreme effects of physical inactivity on body composition and metabolic health. However, these rigorous methods could confound the use of behavioral and cognitive tests performed in rodents (see “Introduction” section) and induce physical inactivity an extreme extent (see “Introduction” section and “A Comparison to Established Models of Inactivity” section above). We show that simple cage modifications, most prominently adjusting the lid to prevent climbing, can be used to model the effects of sedentary behavior.

This is especially useful with regard to studying the effects of reduced physical activity on the brain and cognition, e.g., in the context of animal models for neurodegenerative diseases (Alzheimer’s disease, Parkinson’s disease): our novel method does not share the confounding factors that make established but more restraining inactivity methods less suitable to combine with behavioral tests. In addition, as available inactivity methods induce physical inactivity to quite an extreme extent they may not reflect the more subtle changes induced by sedentary behavior. Thus, in addition to investigating brain and cognition related matters, this new method can be used to assess the effects of inactivity in a number of peripheral disease models (e.g., cardiovascular, cerebrovascular, metabolic and muscular diseases).

## Data Availability Statement

The raw data supporting the conclusions of this manuscript is shown in the (supplementary) figures and tables of this manuscript. All data is stored in the GELIFES data repository and will be made available by the authors, without undue reservation, to any qualified researcher.

## Ethics Statement

This study and all the procedures were approved by the ethical committee for the use of experimental animals (DEC) of the University of Groningen.

## Author Contributions

PR, YH, SH and EZ contributed to the initial conception and design of the study. GD, PD and MH provided further feedback on study design regarding study design according to their specific fields of expertise. PR and YH have performed the behavioral tests and took care of the practicalities until the time of sacrifice. SH performed body composition analysis. YH and PR organized the database. PR performed the statistical analysis. PR wrote the first draft of the manuscript which was revised and contributed too by all authors. SH and GD mainly contributed to sections of the manuscript concerning tissue weights and body composition analysis. PD and EZ mainly contributed to sections of the manuscript concerning anxiety and cognition. MH mainly contributed to sections of the manuscript concerning effects of physical inactivity in humans and statistical analysis. All authors contributed to revision of the complete manuscript, read and approved the submitted version.

## Conflict of Interest

The authors declare that the research was conducted in the absence of any commercial or financial relationships that could be construed as a potential conflict of interest.
