# Supplementary material for: Inducing Physical Inactivity in Mice: Preventing Climbing and Reducing Cage Size Negatively Affect Physical Fitness and Body Composition
Source: Front Behav Neurosci. 2019 Oct 4;13:221. doi: 10.3389/fnbeh.2019.00221 (PMC6797814; doi:10.3389/fnbeh.2019.00221)
Supplement: Supplementary file 1 [file Table_1.pdf]

Supplementary Material

1 Supplementary Figure 1 - Cage size reduction reduces locomotor activity

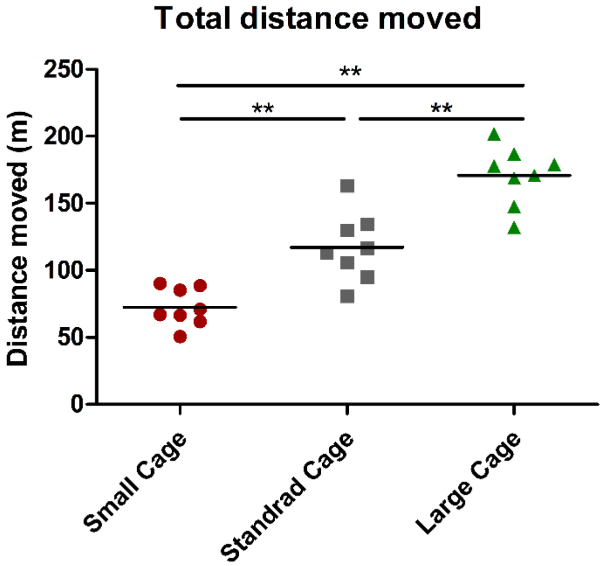

Supplementary Figure 1. Cage size reduction reduces locomotor activity in 4 to 7 month old C57BL6/J mice. Within our facility, it is standard procedure to place individually housed mice in a type 2 macrolon cage (l x w x h ; 33 x 15 x 13 cm). This space was reduced for mice housed in small cages (l x w x h ; 16,5 x 15 x 13 cm) by placing a plexiglass spacer in the middle of the cage. Mice housed in large cages were housed in a type 3 macrolon cage (l x w x h ; 42 x 26 x 15 cm). All cages were covered with a plexiglass lid, which did not allow for lid climbing and which was clear, so video-tracking could be performed using infra-red lights. Animal housing conditions were identical to those described in section 2.2 of the paper. Reducing cage size reduces locomotor activity as compared to standard cage size (Small vs. Standard, $p<0.001$) whereas increasing cage size has the opposite effect (Large vs. Standard ($p=0.006$). An '**' indicates a significant difference for p-values <0.01. Values are mean (stripe) and data points are shown in the scatterplot, n=8 per group. These are repeated measures in a pseudo-randomized design of housing mice in different sized cages for 1 week for each cage size. Distance moved was tracked using Ethovision tracking software (Noldus information technologies) over a 12 hour period during the dark phase at the end of the week. The difference between the means of these 3 dependent variables was tested using a repeated measures ANOVA and subsequent paired T-Tests.

Supplementary Table 1 - Effects of physical inactivity on physical fitness.

**Group**			**Climbing**	**Cage size**	**Climbing x Cage size**	**Small**		**Small – No climbing**		**Standard**		**Standard – No climbing**		**Large**		**Large – No climbing**	
						Mean	SEM	Mean	SEM	Mean	SEM	Mean	SEM	Mean	SEM	Mean	SEM
Balance beam (latency to cross (s))	4mm (angle)	Week -3	#	ns	ns	17.09	0.86	18.76	0.99	17.13	1.83	18.14	2.04	16.43	1.22	19.63	1.17
		Week 5	**	**	ns	21.82	1.39	34.88	2.79	19.43	2.55	26.18	2.39	18.88	1.60	24.34	1.69
		Week 10	**	*	ns	25.70	2.21	39.57	1.60	22.37	3.06	28.83	3.03	25.27	2.97	30.47	2.32
	4mm (horizontal)	Week -3	ns	ns	ns	14.39	0.67	16.26	0.83	15.47	1.33	14.99	1.30	15.65	0.52	17.26	1.07
		Week 5	**	*	ns	17.39	0.80	24.44	1.20	16.52	1.74	19.06	1.50	15.60	1.14	19.38	1.11
		Week 10	*	ns	ns	18.52	1.14	25.00	0.62	17.54	2.13	19.69	1.74	20.89	1.95	22.93	2.27
Grip strength, max (gf)	Forelimb	Week -2	ns	ns	ns	155.40	2.81	149.59	4.41	150.71	3.91	156.11	5.28	153.67	3.73	156.32	3.66
		Week 6	**	ns	ns	127.77	5.04	119.20	3.53	128.48	3.85	117.47	3.93	133.17	3.02	119.40	2.97
		Week 11	ns	ns	ns	117.77	3.23	114.71	2.99	122.67	3.02	118.89	5.62	119.51	4.43	118.79	4.70
	All limbs	Week -2	ns	ns	ns	286.54	6.81	282.25	8.77	288.27	8.20	294.79	10.45	294.69	7.26	287.86	9.18
		Week 6	**	ns	ns	281.54	8.55	241.26	6.11	269.20	11.78	251.97	4.51	291.74	6.24	253.90	8.57
		Week 11	*	#	ns	255.03	6.98	245.34	7.75	273.69	13.09	256.25	7.32	282.76	7.16	257.17	8.29
Inverted screen	Time until fall (s)	Week -1	ns	ns	ns	225.60	32.06	228.20	31.64	219.20	33.83	255.70	52.64	300.30	69.44	169.00	16.90
		Week 7	**	ns	ns	184.30	23.79	63.90	12.14	188.90	29.58	56.60	6.43	247.00	41.39	75.40	17.81
		Week 12	**	ns	ns	162.30	23.63	49.20	7.06	143.60	27.25	55.90	11.04	188.30	32.02	30.20	6.34
	Holding Impulse	Week -1	ns	ns	ns	6001.39	805.67	6074.17	790.73	5991.13	893.77	6891.24	1401.95	8405.08	1969.14	4795.59	487.78
		Week 7	**	ns	ns	5380.51	670.65	1904.37	325.94	5738.04	827.55	1757.27	207.44	7695.52	1347.00	2319.80	560.20
		Week 12	**	ns	ns	5141.18	713.88	1652.59	215.32	4688.91	932.51	1810.99	313.34	6205.67	1139.55	984.83	199.08
Plasma glucose	mmol/l	Sacrifice (week 19)	ns	ns	ns	17.58	0.75	18.09	0.62	15.65	1.34	17.42	.58	18.98	1.55	16.25	1.16

Supplementary Table 1 - Effects of physical inactivity on physical fitness. Main statistical analysis are summarized in the 3rd, 4th and 5th column (two way ANOVA). Capital and lower case letters indicate differences between any of the six groups (one-way ANOVA). '*' and '**' indicate significant differences (p-values <0.05 and <0.01 respectively), a '#' indicates a trend for a difference (p-value <0.10). An 'ns' indicates no significant differences were found. Group sizes are as follows: n=30 per group for Climbing vs. No climbing and n=20 per group for the comparison of different cage sizes (Small, Standard, Large), and n=10 for each of the six distinct housing conditions.

3 Supplementary Table 2 - Effects of physical inactivity on grip strength divided by bodyweight

**Group**		**Climbing**	**Cage size**	**Climbing x Cage size**	**Small**		**Small – No climbing**		**Standard**		**Standard – No climbing**		**Large**		**Large – No climbing**	
					Mean	SEM	Mean	SEM	Mean	SEM	Mean	SEM	Mean	SEM	Mean	SEM
Forelimb max grip strength (gf) / bw (g)	Week 5	**	ns	ns	4.40	0.20	4.00	0.10	4.23	0.18	3.91	0.14	4.33	0.09	3.93	0.09
	Week 10	ns	ns	ns	3.79	0.16	3.52	0.12	3.78	0.13	3.65	0.16	3.65	0.12	3.67	0.15
All limbs max grip strength (gf) / bw (g)	Week 5	**	ns	ns	9.71	0.37	8.13	0.28	8.87	0.46	8.37	0.12	9.52	0.26	8.35	0.21
	Week 10	**	ns	ns	8.21	1.16	7.51	0.58	8.41	1.24	7.88	0.81	8.68	1.00	7.93	0.67

Supplementary Table 2 - Effects of physical inactivity on grip strength divided by bodyweight (indicated by '/ bw'). No differences were found between groups before initiation of the adjustments to induce inactivity. Main statistical analysis are summarized in the 3rd, 4th and 5th column. Capital and lower case letters indicate differences between any of the six groups. An '**' indicates a significant difference for p-values <0.01. An 'ns' indicates no significant differences were found. Group sizes are as follows: n=30 per group for Climbing vs. No climbing and n=20 per group for the comparison of different cage sizes (Small, Standard, Large), and n=10 for each of the six distinct housing conditions.
